# Play deficits in preschool and first-grade school adaptation: a longitudinal study

**DOI:** 10.3389/fpsyg.2026.1879321

**Published:** 2026-07-08

**Authors:** Margarita Gavrilova, Ali Kemal Tekin

**Affiliations:** 1 Faculty of Psychology, Lomonosov Moscow State University, Moscow, Russia; 2 Department of Early Childhood Education, Faculty of Education, Arts and Sports, Western Norway University of Applied Sciences, Bergen, Norway

**Keywords:** play, play deficits, school adaptation, academic engagement, longitudinal study, early childhood

## Abstract

The transition from preschool to primary school is a critical period characterized by adaptation difficulties for a significant number of children. Although play is considered a key context for the development of self-regulation, intrinsic motivation and other abilities necessary for successful school adaptation, its specific role in this process remains understudied. This longitudinal study examined the relationship between play deficits at age 5 and school adaptation outcomes in first grade. The sample comprised 157 first graders (52% boys) who were assessed at age 5 (nonverbal intelligence and ability to play alone) and again at age 7 (the age at which children typically start first grade in the Russian educational system) using the Brief School Adjustment Scale (BSAS). A hierarchical regression analysis revealed that the inability to play alone at age 5 significantly predicted lower overall school adaptation at age 7 (β = −0.107, *p* = 0.034) and, notably, lower academic engagement (β = −0.244, *p* = 0.003), after controlling for the child's sex, age, language proficiency, nonverbal intelligence, maternal education and family income. The effects on other dimensions of school adaptation (school rule-following, psychological well-being and peer relations) were non-significant. These findings are discussed from a cultural-historical perspective, suggesting that solitary play serves as a behavioral marker of the internal plan of action and specifically contributes to academic engagement rather than to rule-following, psychological well-being or social interaction.

## Introduction

The onset of formal schooling is characterized by abrupt changes in the environment; daily routines and the demands and expectations placed upon the child. School introduces new academic and behavioral requirements (Szydlo and Farnsworth, 2023). Furthermore, the transition from preschool to primary school involves changes in peer groups, teachers, activity formats and often the physical building itself (Fukkink et al., 2024; Vitiello et al., 2022). Research underscores the significance of this period for shaping long-term trajectories of learning, mental development and wellbeing. Challenges and stress during this transition can consolidate adverse trajectories and predict later academic outcomes and wellbeing in adolescence (Donaldson et al., 2022; Szydlo and Farnsworth, 2023; Blair, 2002). Notably, aspects of learning considered key to school readiness are most effectively developed through play-based rather than formal pedagogical approaches (O'Sullivan and Ring, 2018).

### School transition and predictors of first-grade adaptation

Research indicates that a considerable proportion of first graders experience challenges in school adaptation. This rate varies depending on the risk group and the criteria used (academic, behavioral, emotional, social problems). The proportion of children with pronounced adaptation problems ranges from approximately 10%−15% (complex academic/behavioral difficulties) to 50%−70% in vulnerable groups (e.g., those with speech disorders) on specific aspects (Taran and Salionovych, 2021). Teachers, parents and psychologists describe a relatively similar level of problematic adaptation in first graders. Many first graders show emotional tension regarding the learning process and school subjects (Taran and Salionovych, 2021). School avoidance, refusal to complete tasks, avoidance of answering, passivity and indifference to lessons may emerge (Taran and Salionovych, 2021). Aggressiveness, irritability, rule violations, disobedience, concentration difficulties and rapid fatigability may also be observed (Demirtaş-Zorbaz and Ergene, 2019; Taran and Salionovych, 2021). Challenges in establishing cooperative relationships, peer rejection, loneliness and victimization can emerge in interactions with classmates (Demirtaş-Zorbaz and Ergene, 2019; Hoglund and Leadbeater, 2004; Ladd and Burgess, 1999). Without timely support, such difficulties can lead to chronic underachievement (Hoglund and Leadbeater, 2004). Thus, identifying early predictors of successful school adaptation is both a theoretical and a practical imperative.

The key factors for a child's adaptation in first grade can be categorized into individual characteristics of the child, the quality of classroom relationships, as well as family support (González-Arratia López-Fuentes et al., 2025). At the child level, successful adaptation is characterized by lower levels of externalizing problems (Demirtaş-Zorbaz and Ergene, 2019; Rademacher et al., 2021), better executive functions (Rademacher et al., 2021; Stankevich, 2024), and social skills (Akçinar, 2013; Rademacher et al., 2021). Within the classroom, closeness and warmth in the teacher-child relationship (Demirtaş-Zorbaz and Ergene, 2019; Hamre and Pianta, 2005), as well as peer acceptance (Demirtaş-Zorbaz and Ergene, 2019), are among the strongest predictors. On the family side, higher socioeconomic status (Akçinar, 2013) and maternal involvement in school life and interest in their child's school experience are associated with better adaptation (Akçinar, 2013). Many of these skills and abilities (self-regulation, rule-following, cooperation with peers and frustration tolerance) can be developed through play (Colliver et al., 2021).

### The role of play in school adaptation: theoretical and empirical foundations

In cultural-historical theory, play is defined by the creation of an imaginary situation, voluntary adherence to hidden rules derived from the adopted role, and acceptance of a social role that prescribes actions and relations. The unity of affect and intellect is central to the developmental power of play (Vygotsky, 1967). Play is conceptualized as the leading activity of preschoolers, through which imagination, volition, self-regulation and social roles are formed, preparing the child for its transition to learning activity (De Moraes, 2023; Broström, 2005; Novlyanskaya, 2025). The primary mechanism of development is the creation of an imaginary situation in which the child acts according to rules, and masters social roles and cultural tools (Ryabkova et al., 2025). According to self-determination theory (Deci and Ryan, 2000), the satisfaction of needs for autonomy and competence during free play also fosters intrinsic motivation which, in turn, should contribute to school readiness and adaptation to the school environment. Consequently, a lack of autonomous (not adult-regulated) play may negatively impact the development of volition, imagination and social skills (Pyle et al., 2022).

Role play (e.g., professions, family, school) is considered a form of practice for mastering social roles and norms, thereby facilitating the adoption of the student role (De Moraes, 2023; Broström, 2005). According to these authors, a rich repertoire of pretend play develops behavioral self-control and impulse management that are necessary for developing correct school behavior. Broström describes play as a “transitory activity” that bridges the systems of play and school learning: advanced forms of play help shape the learning motive and make the child an “agent of their own transition” to school (Broström, 2005). The concept of learning through play in primary school, grounded in developmental science, posits that playful, joyful and socially rich forms of activity ease the transition from preschool to primary school and support the cognitive, social and emotional skills that children need (Parker et al., 2022). Systematic reviews on play-based pedagogies for school transition indicate that play is associated with successful school readiness and the transition to primary school across academic, social and personal domains (Kalinde et al., 2024). For instance, a stable association has been shown between pretend play and social competence (interacting, building relationships, resolving conflicts), which is required for successful adaptation to a new peer group (Nat et al., 2024). Experimental and longitudinal research demonstrates that high-quality social play and “role play with imaginary scenarios” are associated with the growth of executive functions and self-regulation, which are crucial for learning activities (Slot et al., 2017; White et al., 2021; Timmons et al., 2016).

The assumption that solitary play predicts academic engagement needs theoretical justification. According to Elkonin (2005), play develops through several stages. In early stages, play is strongly supported by adults or older children, who propose and maintain the imaginary situation, assign roles and remind the child of them, and state the rules explicitly (Veresov et al., 2021). As the child internalizes the structure of play, it becomes more autonomous. This follows Vygotsky's genetic law of development. What first appears between people later appears inside the child. Solitary play is social play that has been internalized. By the end of preschool age, the child can initiate, sustain, and complete a play session alone. This capacity for solitary play is therefore a marker of what Elkonin called the internal plan of action. It shows that the child has already developed a rich repertoire of play forms, action schemes, imagination and self-regulation. Playing alone requires the child to devise a plot or goal, organize space and materials, maintain the objective and sequence of actions, and control their behavior (Bay, 2024; Gastaldi et al., 2019; Sukhikh et al., 2023). In this sense, solitary play serves as a visible marker of the overall development of play activity, reflecting the maturity of the child's acquired play forms (Cankaya et al., 2023). Research confirms that the more time children aged 2–5 years spend in unstructured quiet play, the higher their self-regulation at ages 4–7, even when controlling for baseline levels and family factors (Colliver et al., 2021). Total time spent playing at home is associated with better self-regulation, and better self-regulation, in turn, is associated with higher early reading and math skills 1 year before starting school (Miller et al., 2022).

Unlike passive pastimes, play requires the child to set goals, plan actions, sustain attention and employ other skills and abilities that will be required in learning activities (Bay, 2024). Moreover, the ability to play alone can be seen as a marker of autonomy and the ability to engage in activity without adult support (Sukhikh et al., 2023; Veresov et al., 2025). For these reasons, deficits in play may have prognostic significance for school adaptation. Yet, despite the arguments outlined above, the link between child's play in preschool and the subsequent success of the transition from preschool to primary school remains underexplored. The present study aims to fill this gap.

### The present study

The aim of this study was to examine the potential effect of play deficits at age 5 on indicators of school adaptation in first grade, after controlling for demographic, socioeconomic, linguistic and cognitive factors. The primary research question was: does the inability to play alone at age 5 predict the composite adaptation score at age 7? Drawing on theoretical perspectives on the developmental value of play within a cultural-historical framework, we hypothesized that the inability to play alone at age 5 would be negatively associated with the overall school adaptation score at the beginning of first grade. This would persist after controlling for the child's sex, age, language proficiency, nonverbal intelligence, maternal education and family income. A secondary research question was: if the effect of play deficits on general adaptation is confirmed, which specific dimensions of school adaptation would be most sensitive to such deficits?

## Methods

### Participants

The final sample comprised 157 first graders (52% boys, 48% girls) participating in the all-Russian longitudinal research project “Growing Together.” The mean age of the children during the school adaptation assessment was 7.6 years (SD = 0.38; in the country in which the study was conducted, children start first grade at age 7). All children had typical development, were born at term, and had participated in the assessment of play activity at age 5. Eighteen teachers (primary school teachers) with higher professional education (bachelor's or master's degree) took part. School adaptation ratings were completed during the first 2 months of the children's entry into first grade, from September–October 2025. Teachers received a detailed information letter about the project and printed forms for each child in their class.

### Procedure

This was a two-wave longitudinal study comprising at age 5 (T1–fall 2023) and school adaptation and demographic data collection at age 7 (T2–fall 2025). At T1, individual assessments of nonverbal intelligence were conducted, and parents completed questionnaires regarding their child's ability to play alone. At T2, teachers completed individual school adaptation forms, including an item on the child's language proficiency, and parents completed a standard demographic questionnaire about maternal education and family income. The study and consent procedures were approved by the Ethics Committee of the Faculty of Psychology at Lomonosov Moscow State University (approval no: 2025/31). Written informed consent was provided by the children's legal guardians.

### Measures

School adaptation was assessed using the Brief School Adjustment Scale (BSAS) (Gavrilova et al., 2026). The scale assesses first graders' school adaptation based on structured teacher observation. The scale consists 16 paired statements describing opposing manifestations of the child's behavior and emotional reactions at school (e.g., items assessing task avoidance vs. task engagement).The items comprise four subscales: Academic Engagement, School Rule-Following, Psychological WellBeing, and Peer Relations. The Academic Engagement subscale (Cronbach's α = 0.94) assesses the child's cognitive activity, engagement in learning, material comprehension, and attitude toward academic tasks. The School Rule-Following subscale (α = 0.90) assesses the ability to follow school rules, accept teacher feedback, and regulate their emotional states. The Psychological WellBeing subscale (α = 0.83) assesses the presence of somatic and emotional manifestations of stress related to schooling. The Peer Relations subscale (α = 0.89) assesses the nature of the child's relationships with their classmates, and social inclusion in the peer group. The overall school adaptation score (Cronbach's α = 0.93) is calculated as the mean of the 16 items, representing an integral assessment of the child's adaptation success. Responses are rated on a four-point Likert scale, with higher scores indicating better adaptation. All four subscales showed acceptable internal consistency (α = 0.83–0.93).

Play deficits were assessed using a single parent-report item: “Please indicate your agreement with the following statement: Your child cannot play alone for even 10 min.” This item was chosen as an indicator of play deficits, reflecting the child's inability to engage in independent, sustained play activity, even for a short period. Responses were rated on a 4-point Likert scale from 1 to 4, with higher scores indicating greater difficulties with play. Consistent with the cultural-historical principles discussed in the Introduction, we avoided direct observation of play. Parents observe their child's play across multiple contexts, providing a more comprehensive picture than a brief laboratory or classroom observation. Moreover, a specific behavioral item focused on a concrete behavior (inability to play alone for 10 min) is less susceptible to subjective interpretation than a global judgement about the child's play competence.

Covariates. Nonverbal intelligence was assessed using Raven's Colored Progressive Matrices. Maternal education and family income were assessed via a parent questionnaire. Language proficiency was rated by teachers on a five-point scale from 1 (very low) to 5 (native-like), then dichotomized for analysis (proficient vs. less proficient).

### Statistical analysis

First, descriptive statistics for all variables and Pearson's correlation coefficients were calculated as a preliminary analysis of associations among the main variables. For the main analysis, hierarchical regression was performed with stepwise addition of variable blocks. Block 1 included basic socio-demographic variables (child's sex and age, maternal education, family income). Block 2 added language proficiency upon school entry. Block 3 added nonverbal intelligence. Block 4 added the primary predictor variable: the inability to play alone for 10 min. The dependent variable was the overall school adaptation score; to test the specificity of the effect, the analysis was then repeated for each of the four subscales (academic engagement, school rule-following, psychological wellbeing, peer relations). The Breusch-Pagan test confirmed homoscedasticity (*p* = 0.838). Variance Inflation Factor (VIF) values were below 1.2, indicating no multicollinearity. Cook's distance showed no influential outliers. Residual non–normality was detected (Shapiro-Wilk *p* < 0.001), which is not problematic for regression with *N* = 157. The statistical significance of each block was assessed by the change in the coefficient of determination (Δ*R*^2^) and the corresponding *F*-test. Multicollinearity was checked for each model (VIF < 5). All analyses were performed using jamovi, with the significance level set at α = 0.05.

## Results

### Descriptive statistics and correlations

Table 1 presents descriptive statistics for all study variables and the associated correlation coefficients. As expected, the inability to play alone at age 5 was negatively associated with the overall school adaptation score (*r* = −0.186, *p* < 0.05), and with its component, academic engagement (*r* = −0.216, *p* < 0.01). Maternal education was negatively correlated with the inability to play alone (*r* = −0.183, *p* < 0.05), indicating that more educated mothers reported fewer play deficits.

**Table 1:** Descriptive statistics and correlations among study variables (*N* = 157).

Variable	M/% (SD)	1	2	3	4	5	6	7	8	9	10	11
1. Sex (% girls)	52.2%	—										
2. Age (months)	92.31 (4.66)	−0.166^*^	—									
3. Maternal education (years)	14.82 (7.26)	−0.010	0.130	—								
4. Family income	2.12 (0.38)	−0.031	−0.126	−0.046	—							
5. Language proficiency (% prof)	88.5%	−0.136	0.019	−0.046	0.201^*^	—						
6. Nonverbal IQ	14.82 (7.26)	0.076	0.211^**^	0.176^*^	−0.080	−0.159^*^	—					
7. Inability to play alone	1.52 (0.81)	−0.084	−0.096	−0.183^*^	0.004	−0.057	−0.043	—				
8. Overall school adaptation	3.44 (0.54)	0.322^***^	−0.077	0.145	0.005	−0.260^***^	0.111	−0.186^*^	—			
9. Academic engagement	3.06 (0.83)	0.309^***^	−0.024	0.114	0.006	−0.352^***^	0.240^**^	−0.216^**^	0.812^***^	—		
10. Behavioral compliance	3.29 (0.78)	0.380^***^	−0.076	0.125	0.011	−0.196^*^	0.055	−0.151	0.868^***^	0.581^***^	—	
11. Emotional wellbeing	3.62 (0.59)	0.175^*^	−0.070	0.212^**^	−0.018	−0.196^*^	0.046	−0.094	0.787^***^	0.456^***^	0.619^***^	—
12. Peer interaction	3.75 (0.47)	0.125	−0.128	0.011	0.013	−0.034	−0.035	−0.073	0.716^***^	0.434^***^	0.520^***^	0.519^***^

For sex: 1 = boy, 2 = girl. For family income and language proficiency, higher values indicate lower income/less proficiency (see Methods). ^*^p < 0.05, ^**^p < 0.01, ^***^p < 0.001.

### Main analysis

Hierarchical regression models were built to examine whether the inability to play alone at age 5 predicted school adaptation at age 7, controlling for the child's sex, age, maternal education, family income, language proficiency and nonverbal intelligence. Table 2 presents the results for the composite adaptation score and for each of the four BSAS subscales. The inability to play alone was added in the final step of each hierarchical regression.

**Table 2:** Hierarchical regression models predicting school adaptation.

Outcome	*R* ^2^	Sex β	Age β	Maternal education β	Family income β	Language β	IQ β	Inability to play alone β	Δ*R*^2^
Overall adaptation	0.202	0.287^*^	−0.007	0.034	0.088	−0.391^**^	0.003	−0.107^*^	0.024^*^
Academic engagement	0.303	0.335^**^	−0.004	−0.001	0.202	−0.958^***^	0.022^*^	−0.244^**^	0.039^**^
Behavioral compliance	0.197	0.535^***^	−0.005	0.048	0.113	−0.395^*^	−0.001	−0.111	0.013
Emotional wellbeing	0.112	0.161	−0.009	0.069^*^	0.033	−0.323^*^	−0.001	−0.045	0.004
Peer interaction	0.034	0.093	−0.012	0.004	0.007	−0.040	−0.002	−0.044	0.005

Each row represents a separate hierarchical regression model. All models controlled for sex, age, maternal education, family income, language proficiency and nonverbal intelligence. Play deficits (inability to play alone) were added in the final step. Δ*R*^2^ represents the change in explained variance after adding the play variable. ^*^p < 0.05, ^**^p < 0.01, ^***^p < 0.001.

The hierarchical regression results indicated that the inability to play alone significantly predicted the overall school adaptation score (β = −0.107, *p* = 0.034, Δ*R*^2^ = 0.024). The demographic and socioeconomic variables (sex, age, maternal education, family income) entered in Block 1 accounted for 12.8% of the variance (*R*^2^ = 0.128, *p* = 0.001). Adding language proficiency in Block 2 increased *R*^2^ to 0.176 (Δ*R*^2^ = 0.048, *p* = 0.003). Adding nonverbal intelligence in Block 3 increased *R*^2^ to 0.178 (Δ*R*^2^ = 0.002, *p* = 0.568). Finally, adding the inability to play alone in Block 4 significantly improved the model, Δ*R*^2^ = 0.024, *F*_(1, 149)_ = 4.56, *p* = 0.034. Children who could not play alone at age 5 showed lower overall school adaptation at age 7. The full model explained 20.2% of the variance in school adaptation (*R*^2^ = 0.202, *p* < 0.001).

The inability to play alone significantly predicted academic engagement only (β = −0.244, *p* = 0.003, Δ*R*^2^ = 0.039), but not school rule-following (β = −0.111, *p* = 0.129), psychological wellbeing (β = −0.045, *p* = 0.437) or peer relations (β = −0.044, *p* = 0.367). Thus, play deficits selectively impact academic engagement, and this effect is more than twice the magnitude of the effect on the composite adaptation score.

## Discussion

The findings indicate that the inability to play alone at age 5 significantly predicted lower overall school adaptation (β = −0.107, Δ*R*^2^ = 0.024) and lower academic engagement (β = −0.244, Δ*R*^2^ = 0.039) at age 7. These effects remained significant after controlling for sex, age, language proficiency, nonverbal intelligence, maternal education, and family income. No significant effects were found for rule-following, psychological wellbeing, or peer relations. A note on epistemology is in order here when interpreting these results. As explained in the Limitations section, the quantitative indicators in this study are best understood as external behavioral markers for testing theory-driven hypotheses at the population level, rather than as direct measures of play development or adaptation processes in the Vygotskian tradition. The effect on overall school adaptation is small, explaining an additional 2.4% of the variance, but such effect sizes are common in longitudinal research with multiple statistical controls. The effect on academic engagement was notably larger, explaining an additional 3.9% of the variance and suggesting a specific influence of play on this aspect of adaptation. The inability to play alone is not presented as a standalone diagnostic criterion but rather as one of several signals that may warrant further observation of the child's play activity. Maternal education was negatively correlated with play deficits (*r* = −0.183, *p* < 0.05). This could reflect genuine differences in play opportunities or response bias. The regression models control for maternal education, so the effect of play deficits is estimated independently of this association.

Play at preschool age was significantly associated with academic engagement in first grade. This may be mediated by at least two psychological processes frequently discussed in the literature on play as a source of development. First, play serves as a natural context for practicing self-regulation skills, which then become the foundation for learning activities and problem-solving in academic contexts (Pyle et al., 2022; Doebel and Lillard, 2023). Self-regulation skills are positively related to task engagement (Bohlmann and Downer, 2015), indicating that the capacity to sustain attention, overcome difficulties and complete tasks, developed in play, transfers to the learning context. Second, play positively impacts the development of intrinsic motivation necessary for developing academic interest and successful learning. In play, the needs for autonomy, competence and relatedness are fully satisfied because the child acts on their own initiative rather than because of external pressure. According to self-determination theory, satisfaction of these needs leads to the formation of a stable intrinsic motivation for learning (Deci and Ryan, 2000).

Challenges in school rule-following, psychological wellbeing and peer relations were not predicted by play deficits. There could be several reasons why play does not directly determine these aspects of school adaptation. First, there is a fundamental difference in the social environment and a discontinuity between preschool and primary education. In contexts in which play activities in preschool and learning activities in school are based on fundamentally different principles, skills acquired in play may be less applicable in school. Play is characterized by voluntariness, flexibility and self-generated rules, whereas learning activity is typically characterized by externally imposed rules not open to discussion (Ma, 2018; Booth et al., 2019). Second, despite the development of self-regulation through play, there may be difficulties in transferring these skills to a new school situation (Savina, 2014). Play creates favorable conditions for developing cognitive and emotional regulation but does not guarantee that such regulation will be used in a new environment, especially under stress (Fomina et al., 2025). Third, the present study focused specifically on solitary play, excluding other forms of play with varying levels of peer or adult involvement (Sukhikh et al., 2025). Specially organized sociodramatic play characterized by planning, role distribution and different scenarios has been shown to positively impact the application of self-regulation skills in everyday life and interaction with others (Veraksa et al., 2024). Likewise, Özcan and Ivrendi (2024) found that high symbolic-complexity sociodramatic play demonstrated significantly better inhibitory control and attention among children.

The study's findings extend the findings of Colliver et al. (2021) on the association between unstructured play and self-regulation, showing that the effect of play is specific to academic engagement rather than to self-regulation-related aspects of school adaptation more broadly. The results of the study are also consistent with the conclusions of Cankaya et al. (2023) on the importance of autonomous play for school readiness. These findings suggest that the ability to play alone can serve as a behavioral marker of the internal plan of action (Sukhikh et al., 2023), which, in turn, will directly affect academic engagement.

### Practical implications

Although this study focused on solitary play, the practical implications vary depending on whether the child can play alone. The inability to play alone should be seen as a marker of underdeveloped play activity rather than an isolated deficit. According to the cultural-historical understanding of play, play activity develops through joint play with peers or adults who model ways of building a plot, distributing roles and adhering to the rules of play. Thus, the practical implications differ depending on whether the child can play alone. When their ability to play alone is underdeveloped, joint play with open-ended materials (e.g., blocks, construction sets, loose parts) that stimulate the imagination and the independent unfolding of play is recommended (Cankaya et al., 2023), with a gradual reduction of adult involvement and the transfer of initiative to the child. Such adult involvement in play has been shown to enhance the quality of play activity and positively impact the development of self-regulation (Veraksa et al., 2024; Sukhikh et al., 2025). Additionally, in contemporary contexts in which online activity often displaces free play (Almazova et al., 2025), these findings may be useful for educating both parents and educators about the value of play.

### Limitations

This study has several limitations. First, the findings should be interpreted considering the epistemological tension between the cultural-historical approach and the use of quantitative methods. We used quantifiable variables to test theoretical hypotheses in a reasonably large longitudinal sample, with full acknowledgment that they do not measure play development or adaptation processes in the strict Vygotskian sense. A similar strategy has been used in other studies drawing on the cultural-historical approach (Colliver et al., 2021; Veraksa et al., 2024). Play deficits were assessed using a single parent-report item. Its use was methodologically justified on cultural-historical and practical grounds (see Methods), but the limitation remains, as single-item parent reports carry the risk of subjectivity and recall bias, and future research should develop qualitative play assessment methods more aligned with cultural-historical approach epistemology. Second, despite the longitudinal design, causal inferences require caution, as unmeasured variables could potentially explain the observed associations. Future studies should include structured observation of play and consider temperamental and relational factors. Usage of different research methods should also be applied such as qualitative approaches. Third, the effect of play deficits on the composite adaptation score was small in magnitude (β = −0.107). In addition, the present study did not distinguish between different types of play, and the proposed mechanisms linking play deficits to academic engagement (self-regulation and intrinsic motivation) were not directly measured. Therefore, these explanations remain speculative, and future research should include direct assessments of these constructs to test mediation formally. Fourth, the sample was drawn from a single educational context (Russia, where school begins at age 7) and is a convenience sample from a longitudinal project. Generalization to other cultural contexts (e.g., countries where school starts at age 5 or 6) or to clinical populations requires caution. Future studies should test the replicability of these findings in diverse samples.

## Conclusion

The inability to play alone at age 5 is associated with challenges in school adaptation in first grade, specifically in academic engagement. These findings refine the cultural-historical understanding of play, considering solitary play as an externally visible marker of the development of the level of play. This shows that solitary play specifically contributes to the mechanisms of independent learning rather than to rule-following, psychological wellbeing or social interaction. The early identification of children unable to play alone may enable targeted support of autonomous play as a factor that contributes to school readiness.

## Data Availability

The raw data supporting the conclusions of this article will be made available by the authors, without undue reservation.
